# Supplementary material for: The Construction and Exploration of a Comprehensive MicroRNA Centered Regulatory Network in Foxtail Millet (Setaria italica L.)
Source: Front Plant Sci. 2022 May 6;13:848474. doi: 10.3389/fpls.2022.848474 (PMC9121102; doi:10.3389/fpls.2022.848474)

T=Seita.4G237600.1_Q=Sit−miR2275d_S=1008

category=2_p=0.979412176375895

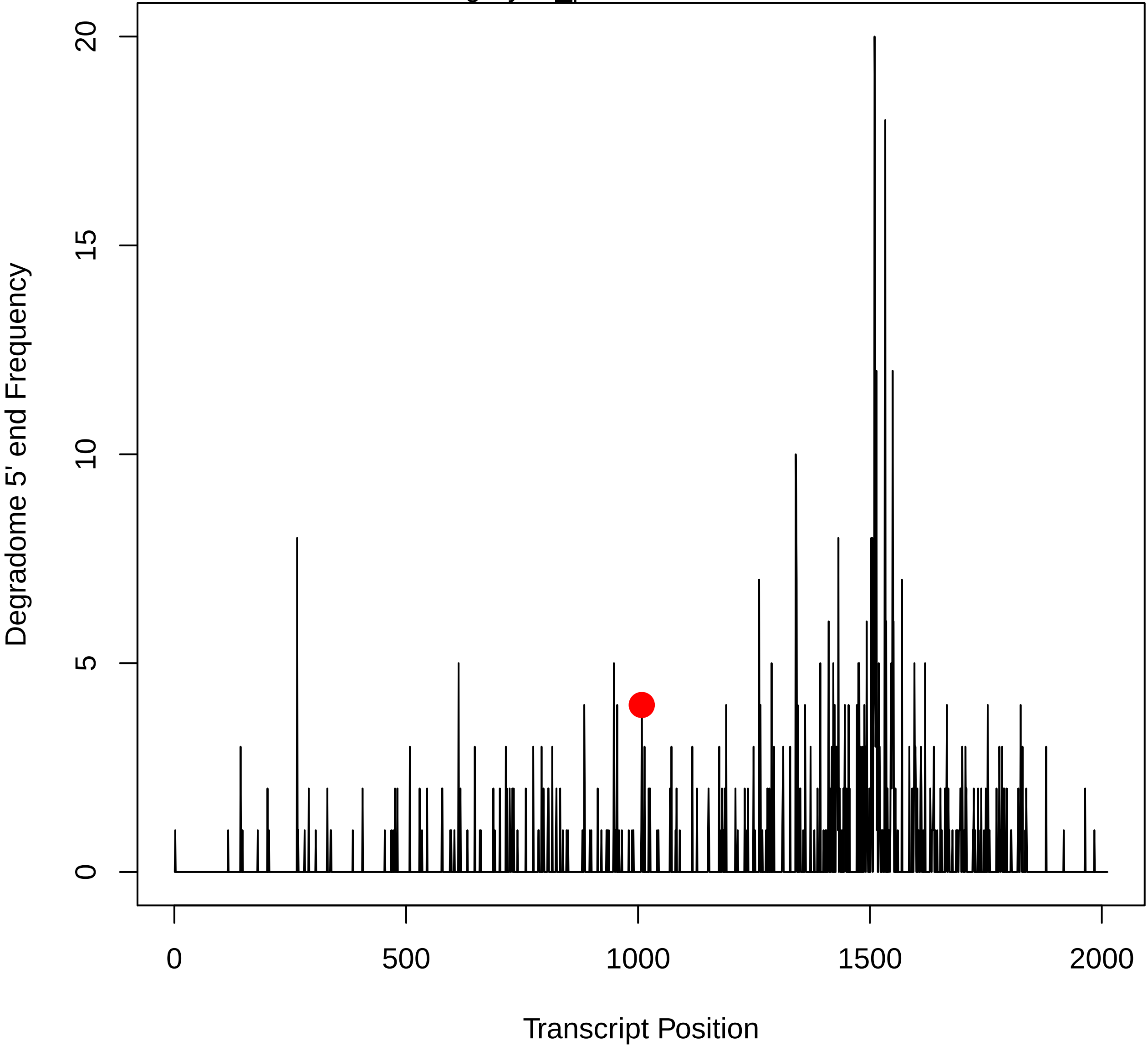

Supplement: Supplementary file 1 [file Data_Sheet_1.zip › Sit-miR2275d_Seita.4G237600.1_1008_TPlot.pdf]
